# The increased motion of lumbar induces ligamentum flavum hypertrophy in a rat model

**DOI:** 10.1186/s12891-021-04203-x

**Published:** 2021-04-06

**Authors:** Baojian Wang, Chunyu Gao, Ping Zhang, Wu Sun, Jingru Zhang, Jinghua Gao

**Affiliations:** 1 Department of Spine, Wangjing Hospital of China Academy of Chinese Medical Sciences, Beijing, China; 2 Department of Pathology, Wangjing Hospital of China Academy of Chinese Medical Sciences, Beijing, China

**Keywords:** Experimental study, Ligamentum flavum, Hypertrophy, Increased motion, Inflammatory factor, Fibrotic factor, Immunohistochemistry, Real‐time PCR, Masson trichrome staining, Lumbar degenerative disease, Rat model

## Abstract

**Background:**

The purpose of this study was to establish a novel rat model for ligamentum flavum (LF) hypertrophy using increased motion of lumbar and to elucidate the etiology of (LFH).

**Methods:**

A total number of 30 male rats were used. The increased motion of lumbar was induced by surgical resection of L5/6 posterior elements (*n* = 15). The other rats underwent a sham operation (n = 15). After 8 weeks, all rats were taken lateral plain X-rays. The LF from L5/6 in both groups were harvested to investigate histological, immunohistological, and real-time PCR analysis.

**Results:**

According to radiological results, the disc height ratio, flexion ratio, and extension ratio were larger in the rats in the experimental group than that of in the sham group. The HE staining showed that the LF thickness in the experimental group significantly increased in comparison to the sham group. The Masson trichrome staining showed that the ratio of elastic fibers to collagen fibers in experimental group was lower than that in the sham group. The protein and gene expression of TGF-β1, TNF-α, IL-1β, and Col 1 were significantly higher in the experimental group than that in the sham group.

**Conclusion:**

A relatively safe, simple, and rapid rat model of LFH using increased motion of lumbar was established. The increased motion of lumbar could lead to high expression of inflammatory and fibrotic factors in LF, causing the accumulation of collagen fibers and decreasing of elastic fibers.

## Background

Lumbar spinal canal stenosis (LSCS) is a common spinal disorder in elder people causing low back pain, gait disturbance, leading to severe disability in the activities of daily living [1]. There are approximately 250,000 to 500,000 LSCS patients in the United States [2]. In these patients with LSCS, protruded lumbar discs, ligamentum flavum hypertrophy (LFH) and bulged facet joints compress the dural sac, cauda equine, or nerve-roots, leading to canal narrowing (stenosis). Because the ligamentum flavum (LF) covers most of the posterior and lateral parts of the spinal canal. The cauda equine or nerve roots can be mechanically compressed by LFH, resulting in spinal symptoms [3]. Numerous investigations have been conducted to clarify the characteristics of LFH in LSCS patients using the tissue harvested from spine surgery [4]. The LFH of human already showed a common histological changes: the loss of elastic fibers and an excessive accumulation of collagen fibers [5, 6]. In addition, molecular overexpression were also found in human hypertrophied LF. The inflammatory factors such as Interleukin-1 (IL-1β), Tumor Necrosis Factor (TNF-α), and fibrogenic cytokines such as Transforming Growth Factor-β1 (TGF-β1) were observed [7–9]. However, whether such factors are causative or merely a consequence of LFH remains unknown. The exact molecular mechanism of LFH has not been revealed yet [10, 11]. Therefore, basic research using an experimental animal model is necessary to clarify its pathomechanism.

Sato et al. [12] reported that LFH could be induced by removel of spinous process, supraspinous ligament, and interspinous ligaments in a rat model. Furthermore, by excising the paraspinal musculature and of rats also resulted in LFH [13]. The above research showed that the destroyed posterior structure of the spine produced mechanical stress on LF. Therefore, we hypothesized that this mechanical stress was directly induced by increased motion of spine and combined resection of muscle, spinous process and other posterior structures could cause more rapid LFH over a shorter time course.

Therefore, in this study, we aimed to establish a rapid rat model for LFH over 8 weeks using resection of more posterior structures. We also intended to investigate the effect of increased motion of lumbar on LF to elucidate the etiology of LFH.

## Methods

### Animals and groups

**Fig. 1:**
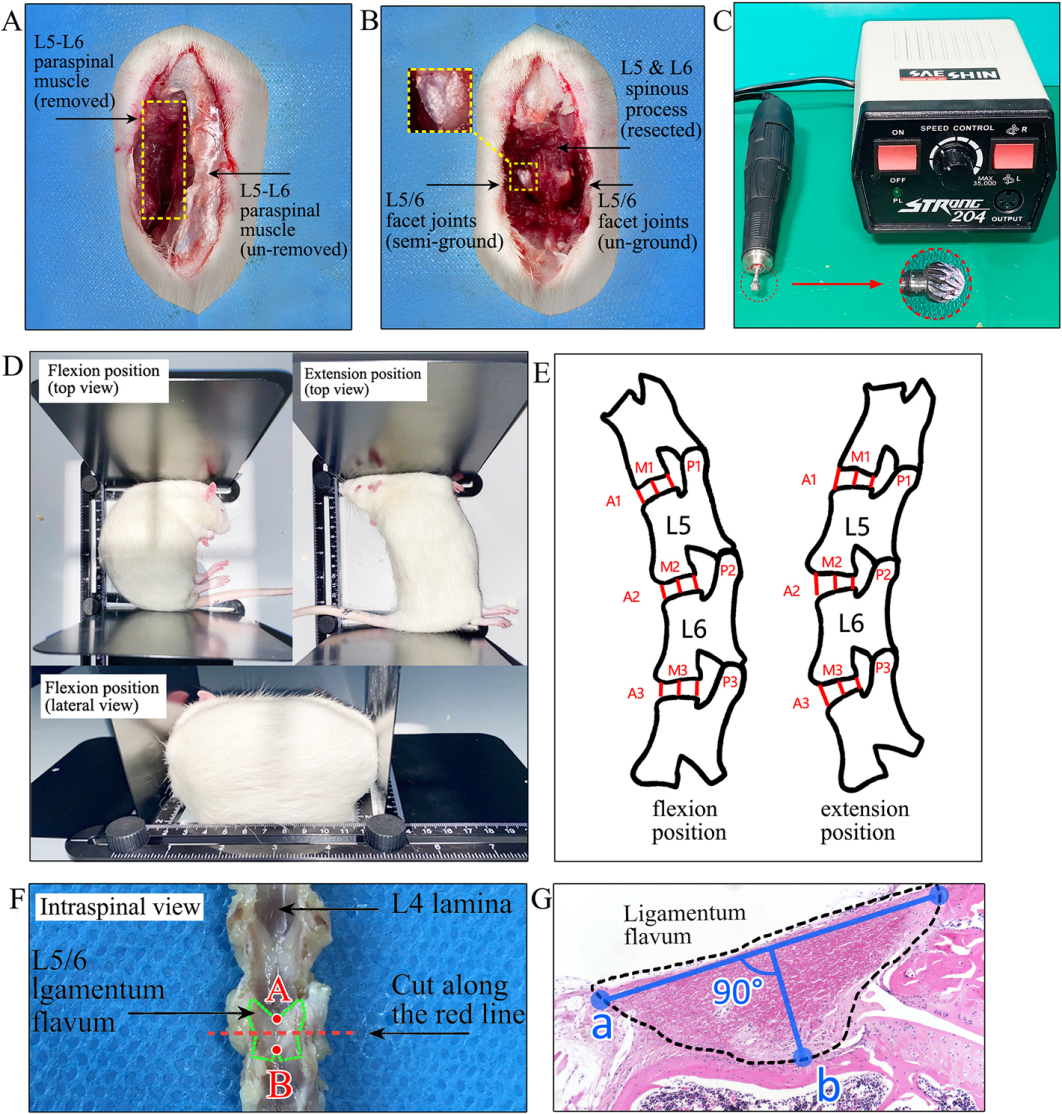
Surgical procedures, radiological methods, cutting level of LF specimen, and measurements of thickness. **a** Removal of L5-L6 paraspinal muscle. **b** Complete resection of L5 and L6 spinous process, semi-grinding of the bilateral L5/6 facet joints. **c** A Grinder and round drill bit used to semi-grind the facet joints. **d** Over extension and flexion position of rats using a retractable holder. **e** Measurement indicators: ①Disc height ratios (DHR) in flexion and extension were calculated as follows: anterior DHR = 2×A2/(A1 + A3), middle DHR = 2×M2/(M1 + M3), posterior DHR = 2×P2/(P1 + P3). ②Flexion ratio (FR) and extension ratio (ER) were calculated as follows: flexion ratio = A2/P2 in flexion, extension ratio = A2/P2 in extension. A anterior, M middle, P posterior. **f** The cutting level of LF after decalcification: the L5/6 posterior lumbar spinal was sliced along the cross plane at the midpoint of upper and lower edges of LF. **g** Measurements of LF thickness and width: blue line “a” represents width, blue line “b” represents thickness

Thirty eight-week-old male Sprague-Dawley rats, each weighing about 250 g were used. During the experiment, all rats were housed in a temperature- and humidity-controlled environment with a 12 h light/12 h dark cycle. All experiments were approved by the animal ethics committee of the Institute of Basic Theory for Chinese Medicine, China Academy of Chinese Medical Sciences (Approval No.2,020,605) and were compliant with NIH guidelines for the humane care and use of laboratory animals. All methods were carried out in accordance with ARRIVE guidelines. During surgical procedures, the rats were anesthetized by an intraperitoneal injection of sodium pentobarbital (50 mg/kg), given injection of antibiotics (20 mg/kg of Cefuroxime Sodium; Medochemie, Cyprus) and then operated aseptically throughout the experiments. They were randomly divided into two groups. The first group (*n* = 15) underwent complete resection of L5 and L6 spinous process, semi-grinding (Grinder, Seashin 204, Daegu, Korea) of the bilateral L5/6 facet joints, removal of whole L5-L6 paraspinal muscle untill lamina exposured (Fig. 1a-c). The second group (*n* = 15) just underwent surgical exposure as a sham operation. All rats were received food pellets and water ad libitum. Two groups of rats were sacrificed at 8 weeks after surgery.

### Radiological analysis

Eight weeks after operation, all rats were taken lateral plain X-rays of the lumbar spine under general anesthesia by an intraperitoneal injection of sodium pentobarbital (50 mg/kg). To ensure the same degree of extension and flexion in all rats, a simple retractable holder was constructed to make sure consistent distance from the head to the tail (Fig. 1d). The motion rang of L5/6 lumbar reflected by disc height ratios (DHR), flexion ratio (FR), and extension ratio (ER) were gauged using Image J 1.50 software (National Institutes of Health) and were compared between two groups [14] (Fig. 1e).

### Human LF samples

Human LF was collected at surgery from 10 lumbar disc herniation (LDH) patients (mean age 41.2 years, range 26–55 years, LF ≤ 4 mm, as non-hypertrophied LF) and 10 LSCS patients (mean age 54.7 years, range 51–71 years, LF>4 mm, as hypertrophied LF) who had undergone decompressive laminectomy. The thickness of human LF was measured at the facet joint level on axial T2-weighed MR images: the vertical distance between dural side and dorsal side of LF [15]. These sections were prepared to Masson trichrome (MT) staining. All procedures were approved by the ethics committee of Wangjing hospital, China Academy of Chinese Medical Sciences (Approval No. WJEC-YJS-2020-009-P002) and performed according to the Declaration of Helsinki. All patients received written informed consent before operation. The LF was collected during surgery after obtaining the informed consent from patients.

### Histological analysis and immunohistological evaluation

The lumbar spinal without any muscles were cut along the coronal plane at the intervertebral foramen to separate them into the anterior and posterior spine column. The posterior lumbar spinal with the LF on the left side were harvested and fixed with 4 % paraformaldehyde phosphate buffer solution for 48 h. Then this posterior lumbar spinal was decalcified in 10 % ethylenediaminetet raacetic acid (EDTA) solution for eight weeks. The L5/6 posterior lumbar spinal were sliced along the axial plane at the midpoint of upper and lower edges of LF (Fig. 1f). These samples were embedded in paraffin and sliced in to 4 μm. This sections were subjected to hematoxylin-eosin (HE) and MT staining. MT staining was to distinguish between collagen fibers (blue) and elastic fibers (red). Ligaments in the human specimens were processed as above without to be decalcified. The Image J 1.50 software was used to measure the area, thickness, width, and the area of collagen or elastic fiber of the rat LF. The measurement methods of LF width and thickness were as follows: The width was a straight line connecting the two ends of LF at dural side (blue line “a” in Fig. 1 g). The longest vertical line crossing line “a” to the dorsal side was the thickness of LF (blue line “b” in Fig. 1 g).

For immunostaining, the sections were retrieved with EDTA retrieval buffers (PH 9.0; Wuhan Boster Biological Technology Ltd., Wuhan, China) at 100 ℃ for 30 min. Endogenous peroxidase activity was quenched in 3 % H_2_O_2_ for 30 min, and endogenous immunoglobulin was then blocked with 3 % bovine serum albumin (Beijing Solarbio Science & Technology, Co., Ltd., Beijing, China) for 30 min. After incubation with TGF-β1 rabbit monoclonal antibody (1:50; Beijing Bioss Biological Technology Ltd., Beijing, China), TNF-α antibody rabbit polyclonal antibody (1:100; Wuhan Boster Biological Technology Ltd., Wuhan, China), IL-1β rabbit monoclonal antibody (1:200; Beijing Bioss Biological Technology Ltd., Beijing, China), and Col 1 rabbit monoclonal antibody (1:100; Beijing Bioss Biological Technology Ltd., Beijing, China) for 2 h, the secondary antibody (1:100; Beijing Bioss Biological Technology Ltd., Beijing, China) incubation was carried out for 2 h. The reaction was visualised using the DAB. The sections were counters-tained using hematoxylin. To perform a quantitative analysis of protein expression for TNF-α, IL-1β, and TGF-β1, the average number of immunopositive cells was counted in 5 fields in each section by 2 independent observers under a light microscope (400×). The results were expressed as average number of immunopositive cells per field in each group. For Col 1, the mean optical density (MOD) of positive cells was analysed using Image J 1.50 software.

### RNA isolation and real‐time PCR analysis

After the rats were sacrificed, the LF of the right side from L5/6 were stored in liquid nitrogen immediately. According to the manufacturer’s instructions, total RNA was isolated from LF tissue using a total RNA preparation kit (Axygen, NY, USA) and cDNA was synthesized from the total RNA using a PrimeScript reverse transcriptase (TAKARA, Shiga, Japan). Relative mRNA expression was determined using RT-PCR with the GoTaq 1-step RT-qPCR system (TAKARA), agarose gel electrophoresis system (BioRad, CA, USA) and qPCR using SYBR premix Ex Taq kit (TAKARA) with ABI Prism 7500 Fast Real-Time PCR system (Applied Biosystems, Foster City, CA). Gene expression was quantified using the 2-^△△CT^ method. GAPDH was used as an internal control. The primer sequences of genes were obtained from Primer Bank. All the primers were synthesized by Servicebio Biotech (Wuhan, China). For real-time PCR, the primers were synthesized as follows: GAPDH, 5’-AAGAAGGTGGTGAAGCAGG-3’ (forward) and 5’- GAAGGTGGAAGAGTGGGAGT-3’ (reverse); TGF-β1, 5’-CCTGTCCAAACTAAGGCTCG-3’ (forward) and 5’-ATGGCGTTGTTGCGGTC-3’ (reverse); IL-1β, 5’-GGGCTGGACTGTTTCTAATGCCTT-3’ (forward) and 5’-CCATCAGAGGCAAGGAGGAAAACA-3’ (reverse); TNF-α, 5’-TTATGGCTCAGGGTCCAACTCTGT-3’ (forward) and 5’-TGGACATTCGAGGCTCCAGTGAAT-3’ (reverse);

Col 1, 5’-AACCCTGGAAACAGACGAACAACC-3’ (forward) and 5’-TGGTCACGTTCAGTTGGTCAAAGG-3’ (reverse).

### Statistical analysis

All statistical analyses were performed with SPSS 22.0 (IBM Corp., Armonk, NY, USA). Student’s t-test were used to compare the radiographical and histological scores between the surgery and sham groups. p values<0.05 were considered significant.

## Results

### Radiological analysis

**Fig. 2:**
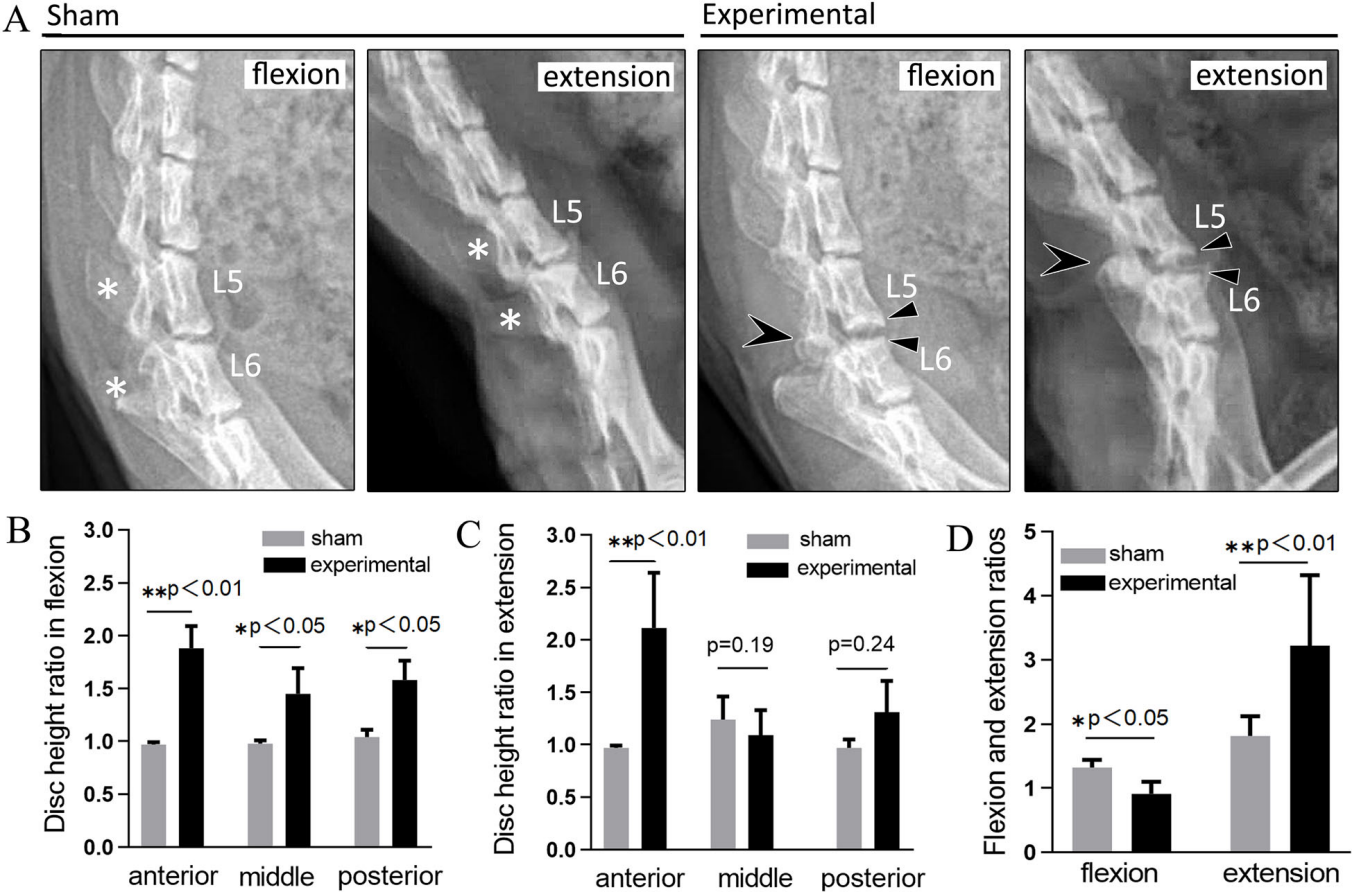
Radiological analysis at 8 weeks postoperatively. **a** Wide disc space and anterior osteophyte formation was found on L5/6 of flexion position and extension position on lateral radiographs. **b** The disc height ratio of the flexion position was larger in the rats in the experimental group than in the rats in the sham groups. **c** The anterior disc height ratio of the extension position was larger in the rats in the experimental group than in the rats in the sham group. **d** The extension ratios were larger in the rats in the experimental group than in the rats in the sham group

According to the radiological results, the rats of experimental group showed wide disc space and bone spurs of the anterior vertebral rims at the L5/6 level (△ in Fig. 2a). The L5 and L6 spinous processes (* in Fig. 2a) of experimental rats were still absent, but the L5/6 facet joints (arrow in Fig. 2a) returned to normal as the other sham rats. No slippage of the vertebral body was seen at at the L5/6 level. The DHR in flexion, ER, and FR were larger in the rats in the experimental group than that of in the sham group (Fig. 2b-d).

### The increased motion of lumbar induced LFH

**Fig. 3:**
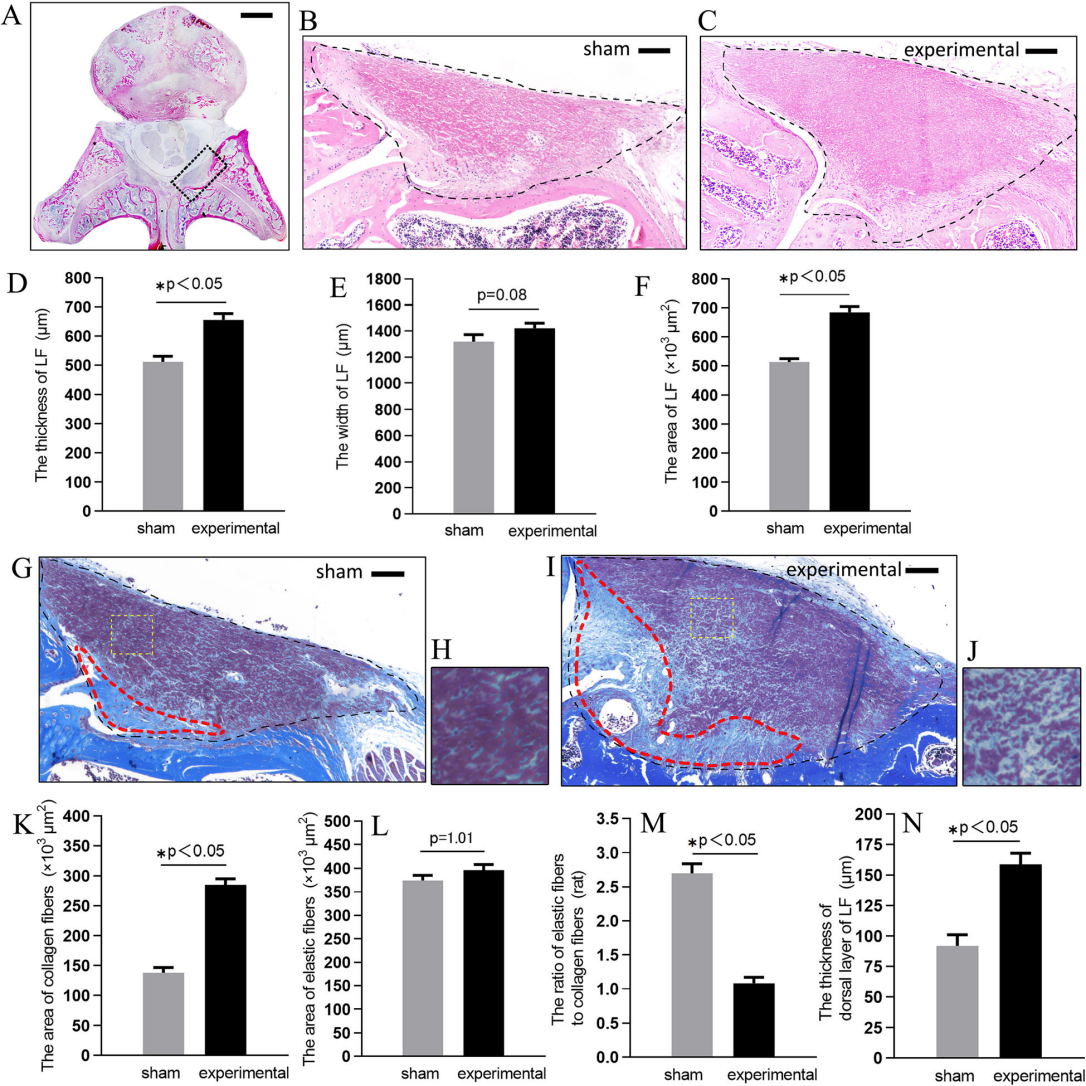
The increased motion of lumbar induced LFH on HE staining and on MT staining. **a** Axial sections of the rat lumbar spine. The cross-sectional area of LF in the sham group and experimental group on HE staining were shown respectively in (**b**) and (**c**). The (**d**), (**e**), and (**f**) Bar graphs showing the thickness, width, and cross-sectional area in the two groups. The cross-sectional area of LF in the sham group and experimental group on MT staining were shown respectively in (**g**) and (**i**). The fibrotic area were in the red circle. The (**h**) and (**j**) were high magnifications of (**g**) and (**i**). The (**k**) and (**l**) Bar graph showed the area of collagen fibers and elastic fibers in the two groups. The (**m**) Bar graph showed the ratio of elastic fibers to collagen fibers in the two groups. The (**n**) bar showed the thickness of dorsal layer of LF. *Scale bar, 500 and 100 μm for low (a) and high (b, c, g, i) magnification images, respectively*

In order to test whether increased motion of lumbar indeed brought about LFH, we compared the L5/6 axial cross-sectional pathological section of all rats. After 8 weeks, The HE staining showed that the thickness of the experimental group was 656 ± 21.4 μm, which was significantly higher than that of the same level in sham group (*p*<0.05). The width of two groups had no distinct difference (p = 0.08). The axial cross-sectional area in the experimental group was about 1.33-fold that of the same level in sham group (*p*<0.05; Fig. 3a-f).

Then we examined the effects of increased motion on the extracellular matrix (ECM) of the rat LF by MT staining. The ratio of elastic fibers to collagen fibers in experimental group was lower than that in the sham group (*p*<0.05; Fig. 3 g-m) .

A blue area was found on the dorsal side of LF in sham group (red circle in Fig. 3 g) and experimental group (red circle in Fig. 3i). This area mainly composed of collagen fibers and had a clear boundary with dorsal layer which mainly composed of elastic fibers. The mean thickness of this enlarged dorsal layer of HF was 159 ± 8.37 μm in experimental group, which was notably thicker than in the sham rat (Fig. 3n).

### The histological analysis of human compared with rat

**Fig. 4:**
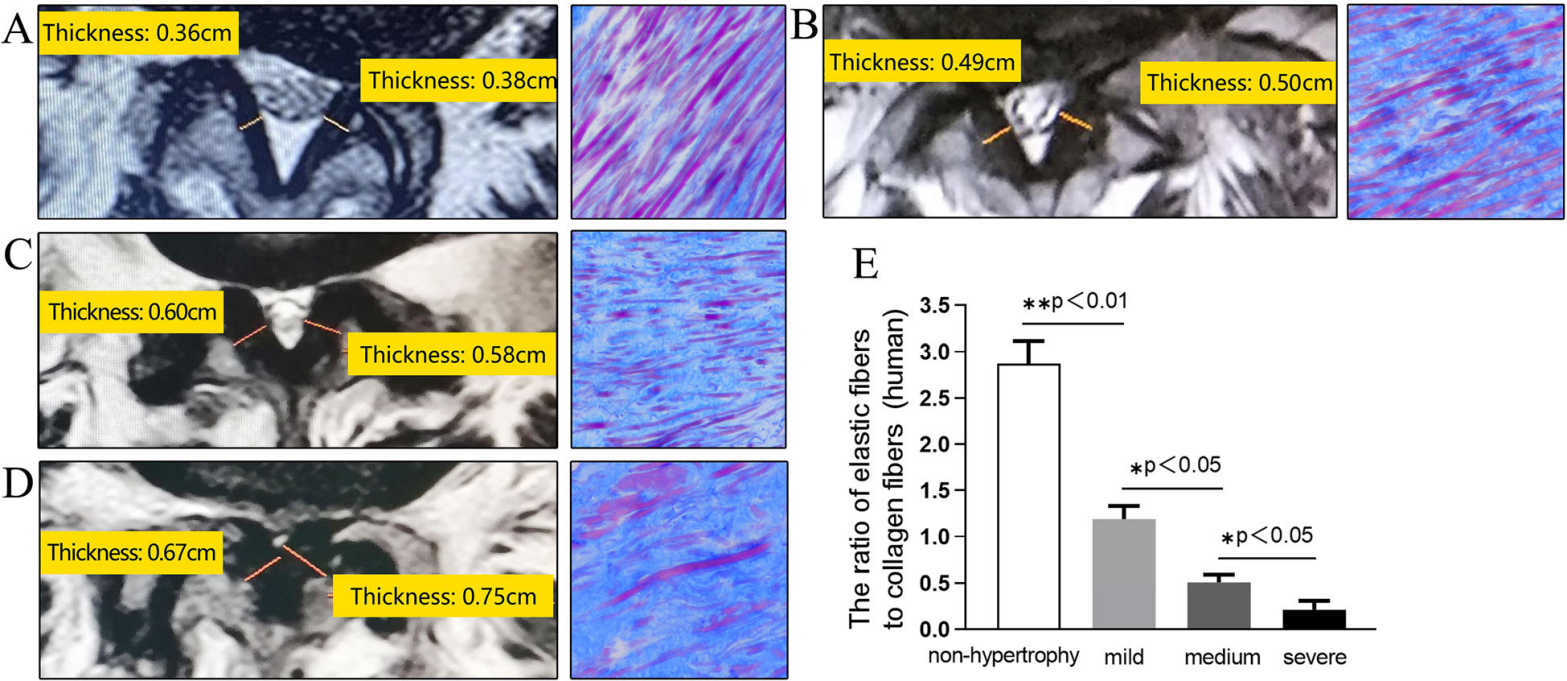
This rat model might be histologically identical to mildly LFH of human. **a**-**d** image showed the MRI and MT staining of human samples from four groups: **a** non-hypertrophy, **b** mild LFH, **c** medium LFH, and **d** severe LFH. The yellow lines indicated thickness of the LF. The (**e**) Bar graph showed the ratio of elastic fibers to collagen fibers in the four human groups

We compared the the severity of LFH in the rat model and that in the human samples. In the human samples, non-hypertrophied LF showed a dense, continuous, and regular bundle of elastic fibers. In contrast, hypertrophied LF showed sparse, fragmented, and irregular elastic fibers on MT staining. The ratio of elastic-to-collagen fibers decreased in hypertrophied human LF. We also found the severity of this histologically change was proportional to the thickness of LF (Fig. 4a-d). Since there was no previous studies involving the grade of LFH, we divided the enrolled patients into three groups: (1) The mild LFH group: 4mm<LF ≤ 5mm; (2) The medium LFH group: 5mm<LF ≤ 6mm; (3) The severe LFH group: LF>6mm (Fig. 4e).

In rat model, the elastic fibers in the sham group were dense which were similar to non-hypertrophied LF of human. Whereas the elastic fibers in the experimental group were sparse, slightly degenerated. The ratio of elastic-to-collagen fibers was 1.19 ± 0.14 in the experimental group (Fig. 3 m), which was similar to human mildly LFH with the ratio of 1.08 ± 0.09 (Fig. 4e).

### The immunohistochemical and PCR analysis

**Fig. 5:**
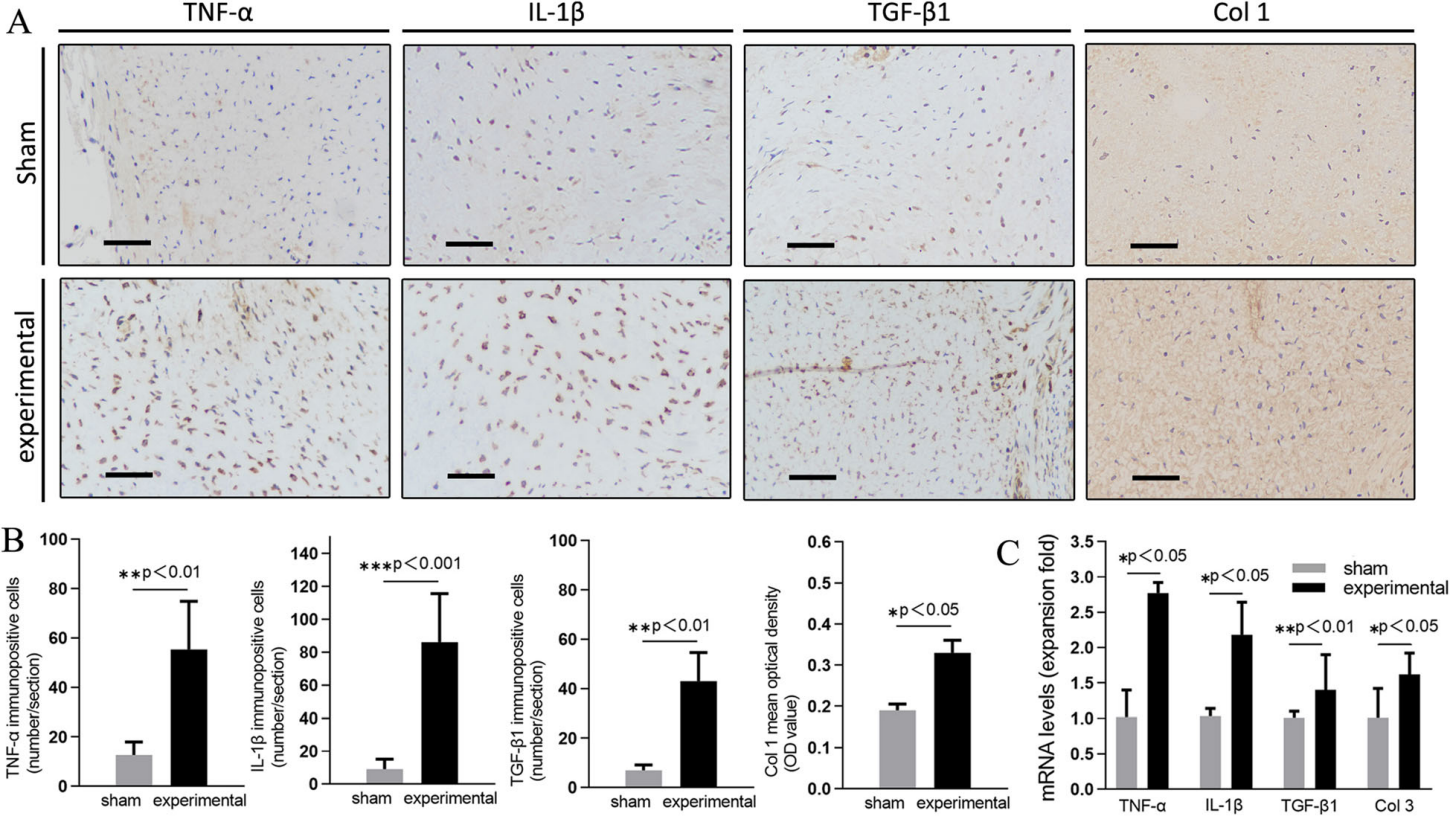
**a** Immunohistochemistry of TGF-β1, IL-1β, and Col 1 in the L5/6 LF of the experimental and sham group at 8 weeks after the surgery. **b** Comparison of immunopositive cells numbers and MOD value. **c** Comparison of mRNA levels (expansion fold). *Original magnification ×400, Scale bar, 50 μm*

Representative specimens of immunohistological staining are shown in Fig. 5a. The LF of L5/6 level in experimental group had significantly larger number of IL-1β, TNF-α, TGF-β1 immunoreactive cells and larger MOD value of Col 1 than that on the same level in sham group (*p*<0.01 or *p*<0.001; Fig. 5b). We also evaluated the gene expression of TGF-β1, TNF-α, IL-1β, and Col 1 in both groups. The PCR analysis demonstrated that the gene expression of TGF-β1, TNF-α, IL-1β, and Col 1 were significantly higher in the experimental group than that in the sham group (*p*<0.05 or *p*<0.01, Fig. 5c).

## Discussion

LFH is one of the major factors of canal narrowing in LSCS. The mechanical stress of lumbar is thought to contribute to LFH [16], but whether this stress can be directly induced by increased motion of lumbar is still uncertain. Therefore, we intend to elucidate the development of LFH over a time course by increased motion of rat lumbar. In this study, we demonstrated that increased motion of lumbar was one of direct factors causing mechanical stress which resulted in LFH.

Previous study reported that intervertebral mechanical stress induced LFH with posterolateral fusion instrumentation in a rabbit model over 16 weeks [17]. saito et al. [18] established a mouse model of LFH using a loading device for applying consecutive mechanical flexion-extension stress to lumbar over 12 weeks. However, the successful establishment of above models will take a long time and requires special devices or equipments. The LFH could also be induced by removel of spinous process, ligament [12], or paraspinal muscles [13]. Therefore, we tried to destroy spinous process with ligaments, paraspinal muscle, and facet joints to concentrate much more stress on a certain lumbar segment, which was expected to induce more rapid LFH within 8 weeks. A part of fibers in LF connect to interspinous ligament or facet joint capsule according to human anatomy. Dissimilarly, the LF of rats are mainly distributed on the left and right facet joints (Fig. 3a). Moreover, we measured the thickness of LF of both sides on axial plane, not on the central sagittal plane. So the resection of interspinous ligament might have limited influence on LF and final results. In addition, to aviod the direct invasion of LF fibers on facet joint, we merely half ground the facet joints, not total grinding or even total resection. Consequently, this kind of model was relatively simple, rapid and safe.

The radiographical analyses showed wide disc space, increased range of motion and bony spur formation. Similar to our data, Fukui et al. [14] found that increased disc height and endplate irregularities were observed at surgery segment after lumbar facetectomy in the rat. In contrast, the disc height decreased after lumbar facetectomy in the recent mouse IDD model [19]. The discrepancy between these models may be due to the difference of surgical procedures, position of X-ray (dynamic position VS. normal position), observation time points, or that of species (rat VS. mouse).

To assess whether the rat was a feasible experimental animal model for the study of LFH, we evaluated histological results of the rat lumbar spine. In the axial sections of the lumbar spine, HE staining demonstrated that the rat LF was located between the dural sac and facet joints. In the sagittal sections, the LF ran between adjacent laminas. These histological features were very similar to those of human LF. Furthermore, the normal LF in humans is a well-defined elastic structure with 75 % elastic fibres and 25 % collagen fibres [6]. In this study, the density of elastic fibres with MT stain in sham group was almost equal to that of the normal LF in humans. Based on these findings, the structure of the LF in rats was similar to that in humans. Therefore, using a rat model to examine the pathomechanism underlying LFH for the study was considered reasonable. Previous studies had demonstrated that the proportion of collagen fibers was accordingly increased when LF hypertrophied [5, 6]. Our experiment showed similar results. In addition, the thickness and area of LF were higher than that of the same level in sham group. These results indicated the successful establishment of a LFH rat model by increased motion of lumbar. Moreover our rat model might be histologically identical to mildly LFH of human.

The epiligament constitutes the surface layer of ligaments and consists of woven bundles of collagen fibers [20]. Bray et al. [21] also found that medial collateral ligament (MCL) hypertrophy of the epiligament induced MCL hypertrophy in the animal knee instability model. Thus, the epiligament can be considered to play a major role in ligament hypertrophy. In our study, the increased motion of lumbar probably caused the thicken of the dorsal epiligament. This histological finding was similar to the human samples from the LSCS patients [12]. Sairyo et al. [16] previously reported that mechanical stress at the dorsal aspect was about 5-fold higher than that at the dural aspect of the LF. Flexion is the most important motion for inducing mechanical stress in the LF. Higher mechanical stress at the dorsal aspect may induce micro injury in daily activities. During the process of micro injury healing, a thick fibrotic mass may be produced.

The increased expression of TGF-β1, IL-1β, TNF-α and Col 1 was similar to the previous results in human hypertrophied LF [8, 9]. Inflammation-related gene expression such as COX-2, TNF-α, and IL-1, -6, -8, -15 were found in the human LF. Its expression showed weak positive linear correlation with the thickness of ligament [7]. Among the proinflammatory cytokines involved in degenerative spinal diseases, IL-1β and TNF-α are considered the major players [22]. Otherwise accumulation of fibrosis (scarring) caused hypertrophy of the ligamentum flavum. In fibrotic diseases of several organs, TGF-β1 has been reported to be an important disease related factor for collagen production and deposition [2]. In hypertrophied LF of humans, macrophages as well as vascular endothelial cells but not only fibroblasts showed a strong expression of TGF-β1 [23]. Sairyo et al. [22] reported that the expression of TGF -β1 mRNA was higher in the early stage of the LF degeneration, but not in the later stage. Its expression decreased as the ligamentum flavum thickness increased. The over expression of TGF- β 1 might not completely cover the whole process of LFH, which multiple factors involved. The expression of TGF -β1 is still controversial in present studies. Therefore, we should continue to explore the expression of this factor regarding to different stages of LFH in the future.

There are still several limitations in our study. Firstly, we should note the facts that LF are not the same in rat and human in terms of their size, nutrition, and the biomechanical stress that they receive (less axial compression or torsion) [24]. These differences should be considered in judging the results obtained from the present model. While recent work used patient-derived LF cells as an in vitro three-dimensional (3D) model for studying LFH which better recapitulated the cell-cell and cell-matrix interactions occurring in LF tissue [25]. Similarly, this kind of model can not effectively simulate the complex mechanical, chemical, nutritional and metabolic environment inside the spine. Otherwise the source of the primary LF sample greatly affects the quality of the formation of cells clusters which makes it still have some limitations in mechanism exploration and new drug development. Secondly, there was no previous studies involving the grade of LFH, we divided the LSCS patients into three groups which was a small sample size collected from 10 humans. Therefore, the availability of this section may be limited. Third, we should carry out Western Blot analysis to further verify the protein expression according to TGF-β1, IL-1β, TNF-α and Col 1. Nevertheless, we believe that our model established in this study partly showed the pathological features of human hypertrophied LF, and is very helpful for us to understand its pathological process and mechanism.

## Conclusions

In conclusion, we established a relatively safe, simple, and rapid rat model of LFH using increased motion of lumbar. In addition, increased motion of lumbar could lead to high expression of inflammatory and fibrotic factors in LF, causing the accumulation of collagen fibers and decreasing of elastic fibers.

## Data Availability

The datasets used and/or analysed during the current study are available from the corresponding author on reasonable request.

## Abbreviations

LSCS: Lumbar spinal canal stenosis
LF: Ligamentum flavum
IL-1β: Interleukin-1
TNF-α: Tumor Necrosis Factor
TGF-β1: Transforming Growth Factor-β1
Col 1: Collagen 1
DHR: Disc height ratios
A: Anterior
M: Middle
P: Posterior
MRI: Magnetic resonance imaging
HE: Ematoxylin-eosin
MT: Masson trichrome
MOD: Mean optical density
ECM: Extracellular matrix
IDD: Intervertebral disc degeneration
MCL: Medial collateral ligament
